# A case report of *Nocardia *spp*.* infective endocarditis in an injection drug user

**DOI:** 10.1186/s12879-021-06541-6

**Published:** 2021-08-19

**Authors:** Chukwunyelu Enwezor, Courtney L. Russ-Friedman, Zachary P. Gruss, Adam Murphy, Elizabeth L. Palavecino, Niyati Jakharia

**Affiliations:** 1 Department of Internal Medicine, Section of Infectious Diseases, Wake Forest Baptist Medical Center, 1 Medical Center Blvd, Winston-Salem, NC 27157 USA; 2 Department of Pharmacy, Wake Forest Baptist Medical Center, Winston-Salem, NC USA; 3 Department of Pathology, Wake Forest Baptist Medical Center, Winston-Salem, NC USA; 4 Department of Internal Medicine, Division of Infectious Diseases, Stanford University Hospital, Palo Alto, CA USA

**Keywords:** *Nocardia *spp., Endocarditis, Injection drug use, Case report

## Abstract

**Background:**

Nocardia-related endocarditis is rare. Intravenous drug use with nonsterile injection practices is a potential risk factor for nocardia infection. Disseminated nocardiosis with endovascular involvement is rarely reported in immunocompetent individuals.

**Case presentation:**

A 54-year-old male was diagnosed with infective endocarditis due to *Nocardia asteroides* with septic emboli in the brain and spleen. The use of a matrix-assisted laser desorption ionization-time of flight mass spectrometry (MALDI-TOF MS) rapid diagnostic system was beneficial in identifying the causative organism. He was empirically treated with combination therapy consisting of three antibiotics. Antimicrobial susceptibility testing indicated that all three antibiotics had favorable minimum inhibitory concentrations (MICs). Due to his clinical status, he was not a surgical candidate. Patient passed away after discharge to hospice.

**Conclusions:**

This case demonstrates unique challenges in the identification, diagnosis, and management of Nocardia-related infective endocarditis. A detailed history of injection practices should guide clinicians in assessing the risk for environmental pathogens. Valvular surgery and combination antibiotic therapy should be recommended for all eligible patients to improve the chances of survival.

## Background

The diagnosis of Nocardia-related infective endocarditis is rare, especially in the immunocompetent population. Nonsterile injection practices, such as needle washing, among persons who use drugs (PWUD) increase the risk of infection with atypical environmental pathogens. Traditionally, microbiologic identification of *Nocardia* spp*.* has been difficult due to misidentification and prolonged incubation requirements. The optimal treatment regimen for Nocardia-related infective endocarditis involves surgery for source control and combination antibiotic therapy. We report a 54-year-old male who was diagnosed with infective endocarditis due to *Nocardia spp.* and treated with combination of antibiotics.

## Case presentation

A 54-year-old incarcerated male with a history of hypertension, alcohol abuse, injection drug use (IDU), and hepatitis B infection presented with fever, right-sided weakness and recurrent falls for two weeks. The patient reported injecting methamphetamine into his veins one month prior to admission and using water to clean his needles.

His vital signs were significant; he had a temperature of 99.9 F; tachycardia, with a heart rate of 120 bpm; and his blood pressure was 112/66 mmHg. The physical examination revealed bilateral weakness with pronator drift noted in the right upper extremity and dysarthria. Due to a high suspicion of stroke, magnetic resonance imaging of the brain was performed. It showed late subacute infarcts involving the left middle cerebral artery and right frontal subcortical white matter, with associated petechial hemorrhage. These findings suggested cardioembolic stroke. Transthoracic echocardiography showed severe aortic stenosis and a right to left interatrial shunt. Computed tomography of the chest, abdomen and pelvis revealed splenic infarcts.

Laboratory data revealed mild leukocytosis, with a white blood cell (WBC) count of 11.5 × 10*3/µL (reference range: 4.8–10.8 × 10*3/µL); thrombocytopenia, with a platelet count of 78 × 10*3/µL (reference range: 160–360 10*3/µL); and mild transaminitis, with an aspartate aminotransferase (AST) level of 47 IU/L (reference range: 5–40 IU/L) and alanine aminotransferase (ALT) level of 60 IU (reference range: 5–50 IU/L). Creatinine was 0.68 mg/dL (reference range: 0.5–1.50 mg/dL) and blood urea nitrogen (BUN) of 15 mg/dL (reference range: 8–24 mg/dL).

During hospitalization, he became febrile, with the body temperature reaching 102 F. Four sets of bacterial blood cultures were obtained. They were incubated with the BACTEC FX blood culture system (BD, Spark, MD). The patient was started on broad spectrum antibiotics including vancomycin and cefepime. Two sets of blood cultures turned positive on days two and three of incubation and showed gram-positive rods on examination with Gram stain. The subculture plates from these sets showed growth after only 48 h of incubation, when pinpoint white, dry colonies were observed on the aerobic blood agar plates. The organism was identified as *Nocardia *spp. by MALDI Biotyper CA System (MBT-CA; Bruker Daltonics, Billerica, MA) software version 3.2. 16 using pretreatment with formic acid according to the manufacturer’s instructions for clinical applications.

Due to cerebral and splenic emboli, there was high suspicion of endocarditis. Transesophageal echocardiography showed a heavily thickened and degenerated aortic valve (AV) with concomitant vegetation and abscess formation on the left coronary cusp resulting in dehiscence, perforation, and AV insufficiency. No other valves were involved. The patient’s antibiotics were switched to trimethoprim-sulfamethoxazole (TMP-SMX) 15 mg/kg/day IV divided in four doses, amikacin 21 mg/kg IV every 24 h, and imipenem-cilastatin 500 mg IV every 6 h. This antibiotic regimen was started on day 7 of admission. Repeat blood cultures remained negative. Cardiothoracic surgery was considered and a pre-surgical work-up was recommended, including digital subtraction angiography (DSA) and left heart catheterization (LHC), due to his history of tobacco use. LHC was not performed due to the patient’s worsening renal function to avoid contrast agent exposure. He ultimately developed renal failure requiring hemodialysis on the 19th day of hospitalization [Creatinine was 3.76 mg/dL, BUN of 54 mg/dL with an estimated glomerular filtration rate (eGFR) of 17 (reference range: > 60 ml/min/1.73 M*2)]. The patient’s clinical status continued to decline, with hypotension requiring vasopressors, worsening respiratory status requiring intubation, and acute renal failure requiring continuous renal replacement therapy (CRRT). Antimicrobial susceptibility testing, performed at a Nocardia reference laboratory using broth microdilution and CLSI breakpoints, showed that the organism was susceptible to amikacin, ceftriaxone, imipenem-cilastatin, linezolid, moxifloxacin, TMP-SMX, and tobramycin (Table 1). He was deemed not eligible for valvular surgery due to his worsening clinical status. After family discussion, he was transitioned to outpatient hospice care, and all antibiotics were discontinued. Patient passed away a day after discharge to a hospice care facility.

**Table 1:** Reported cases of endocarditis due to *Nocardia* species

References	Age (y)/sex	Predisposing risk factor	Site of involvement	*Nocardia* species		Antimicrobial treatment (Duration)	Surgery	Outcome
Allevato et al*.* [8]	53/M	PAV	RAA	*N. asteroides*		Streptomycin, tetracycline, vancomycin, sulfonamides	N/A	Died
Antony et al*.* [9]	74/F	HTN, valvular heart disease, HLD	NMV	*N. asteroides*		Imipenem, ceftriaxone (6 weeks), TMP-SMX (6 months)	MVR	Survived
Antonovich et al*.* [10]	83/F	COPD, MVP, breast & endometrial cancer, systemic steroids	NMV	*N. asteroides*		TMP-SMX	N/A	Survived
Cargill et al*.* [11]	85/F	Ischemic heart disease, COPD, polymyalgia rheumatic, systemic steroids	NMV	*N. cyriacigeorgica*		Vancomycin, pip/tazo, imipenem, doxycycline	N/A	Died
Castelli et al*.* [12]	36/M	Liver transplant	NMV	*N. species*	Ceftriaxone, vancomycin, TMP-SMX		MVR (× 2)	Survived
Daikos et al*. *[13]	61/F	PAV	PAV	*N. asteroides*	Imipenem/cilastatin, amikacin (2 months), TMP-SMX (4 months)		N/A	Survived
Dhawan et al*.* [14]	46/F	N/A	NMV	*N. species*	PCN, nafcillin, gentamicin, TMP-SMX, imipenem/cilastatin		MVR	Survived
Eigel et al*.* [15]	61/M	PAV	PAV	*N. asteroides*	Amikacin, imipenem/cilastatin, cefotiam (3 weeks)		AVR	Survived
Eigel et al*.* [15]	65/M	PAV	PAV	*N. asteroides*	N/A		N/A	Died
Ertl et al*.* [16]	61/M	PAV	PAV	*N. asteroides*	Amoxicillin, mezlocillin, tobramycin, sulphadiazine, amikacin, amoxicillin-clavulanate, imipenem (3 weeks), TMP-SMX		AVR	Survived
Falk et al*.* [17]	64/F	PAV	PAV	*N. asteroides*	Ampicillin, gentamicin		N/A	Died
Gupta et al*.* [18]	53/F	History of breast cancer	NAV	*N. species*	Meropenem, amikacin, TMP-SMX, linezolid (8 months)		AVR	Survived
Hazim et al*.* [19]	64/M	Heart transplant, CKD	NTV	*N. asteroides*	TMP-SMX, imipenem/cilastatin, ceftriaxone, minocycline, linezolid (7 months)		N/A	Survived
Jackson et al*.* [20]	39/M	Splenectomy, tobacco use, HCV, IVDU	NTV	*N. francinia*	Meropenem, vancomycin, TMP-SMX		N/A	Lost to follow-up
Kuretski et al*.* [21]	58/F	History of oral cancer, COPD, PFO repair, tobacco use	NMV	*N. farcinica*	Ceftriaxone, meropenem, amikacin, ciprofloxacin		MVR	Survived
Majeed et al*.* [22]	72/M	Lymphoma	NMV	*N. kroppenstedtii*	Meropenem, TMP-SMX, levofloxacin, linezolid, ceftriaxone, minocycline, ciprofloxacin		N/A	Died
Vlachakis et al*.* [23]	34/F	PMV, rheumatic heart disease	PMV	*N. asteroides*	N/A		N/A	Died
Watson et al*.* [24]	39/M	IVDU	NAV	*N. asteroides*	Meropenem, ceftriaxone, amikacin, cefpodoxime, TMP-SMX (6 months)		AVR	Survived

AVR, aortic valve replacement; CKD, chronic kidney disease; COPD, chronic obstructive pulmonary disease; HCV, Hepatitis C virus; HLD, hyperlipidemia; HTN, hypertension; IVDU, intravenous drug use; MVP, mitral valve prolapse; MVR, mitral valve replacement; NAV, native aortic valve; NMV, native mitral valve; NTV, native tricuspid valve; PFO, patent foramen ovale; Pip/tazo, piperacillin and tazobactam; PCN, penicillin; PAV, prosthetic aortic valve; PMV, prosthetic mitral valve; PV, prosthetic valve; RAA, root of ascending aorta; TMP-SMX, trimethoprim sulfamethoxazole

## Discussion and conclusion

We report a 54-year-old male with IDU presenting with aortic valve endocarditis due to *Nocardia *spp. This case is unique, as it is the first published report of infective endocarditis caused by *Nocardia spp.* secondary to nonsterile injection practices in an immunocompetent host. The source of entry of this infection was likely secondary to nonsterile injection practices.

*Nocardia* spp. are ubiquitous environmental saprophytes occurring in soil, organic matter, and aquatic habitats. Microscopically, *Nocardia* spp. are characterized as gram-positive, beaded, branching, weakly acid-fast rods. Historically, some *Nocardia* spp. may be difficult to detect on standard blood culture media due to their prolonged incubation requirement 2–4 days) [1, 2]. The isolate could be recovered on fungal cultures as these are incubated for 4–6 weeks. *Nocardia* spp. are often misidentified as other gram-positive rods, which also leads to a delay in identification. The increased use of rapid diagnostic laboratory tests, such as MALDI-TOF MS, has significantly decreased the time to identification of this bacteria once it grows on solid agar media, as in this case [3]. However, the complex and continuously changing taxonomy of this genus, makes difficult, particularly for *Nocardia *spp.*,* to confirm the identification as different system may have a different library (2). In this case, the organism was identified as *Nocardia asteroides* with a score of 2.24, which is considered a good identification score. Disseminated Nocardia infection is most commonly diagnosed in patients with underlying immunocompromising conditions and is associated with a high rate of mortality (26%) [4–6]. The common sites of infection include the lungs, central nervous system, skin and subcutaneous tissues. The joints, bone, and heart are less commonly involved [7].

We conducted a literature search of English language reports in the PubMed, Ovid and EBSCO databases using the terms “endocarditis” and “nocardia”. We describe the risk factors and outcomes of 18 cases of Nocardia-related infective endocarditis in Table 2. The median patient age was 61 years. *Nocardia asteroides* was the most commonly reported species (61%), followed by *Nocardia farcinia* (11%) and other *Nocardia* spp. (28%) [8–24]. The most common risk factors in the reported cases were a prosthetic aortic valve (33%) [8, 13, 15–17], cancer (22%) [10, 18, 21, 22], and chronic obstructive pulmonary disease (COPD) (17%) [10, 11, 21]. Other pertinent risk factors included solid organ transplantation (11%) [5, 19], systemic steroid use (11%) [10, 11], and rheumatic diseases (11%) [11, 23]. Notably, the only risk factor observed in this patient was IDU, which has been reported in only two other cases [20, 24]. Certain practices, such as needle sharing, licking, washing and nonsterile injection methods, may increase the risk of infections from environmental pathogens [25]. It is important to emphasize and educate patients about sterile practices to prevent severe infection.

**Table 2:** Minimum inhibitor concentration results of antibiotics tested on the *Nocardia spp* isolate

Antibiotics	Minimum inhibitory concentration (MIC) (mcg/mL)	Interpretation
TMP-SMX	< 0.25/4.75	S
Linezolid	2	S
Ciprofloxacin	2	I
Imipenem	< 2	S
Moxifloxacin	0.5	S
Amoxacillin-clavulanic acid	> 64/32	R
Amikacin	< 1	S
Ceftriaxone	8	S
Doxycycline	2	I
Minocycline	2	I
Tobramycin	< 1	S
Clarithromycin	8	R

MIC analysis was performed at Wake Forest Baptist Hospital (WFBH) laboratory by broth microdilution and interpretation of MICs was determined in accordance with CLSI guidelines

S, susceptible; I, intermediate; R, resistant

The paucity of data regarding Nocardia-related infective endocarditis has hindered the ability of clinicians to determine the optimal treatment regimen and duration. Eleven patients (Table 2) received at least three drugs for infective endocarditis treatment [8, 9, 11, 13, 15, 18–22, 24]. Eight of these patients survived [9, 13, 15–21, 24]. The three patients who died did not undergo valvular surgery [8, 11, 22]. Of the seven patients [10, 12, 14–17, 23] who received less than three antibiotics, four patients survived [10, 12, 14, 16]. Irrespective of the number of antibiotics used to treat their infection, all eight patients who underwent valvular surgery survived [9, 12, 14–16, 18, 21, 24]. These findings indicate that valvular surgery is imperative for source control and improving outcomes in patients with a bacterial infection that is very difficult to treat. The cases in Table 2 had widely variable treatment durations, ranging from 3 weeks to 8 months. Despite the inconsistence with treatment duration and clinical success, it may be prudent to treat endocarditis due to Nocardia similarly to other serious infections caused by *Nocardia* spp., with combination antibiotic therapy for several months. Two agents commonly used to treat serious Nocardia infections, TMP-SMX and amikacin, have been associated with nephrotoxic effects, warranting close observation of renal function and therapeutic drug monitoring throughout the treatment period [7, 26]. Unfortunately, the use of concomitant nephrotoxic medications combined with hypoperfusion due to cardiogenic shock led to renal failure in this patient. Thus, his tenuous status lead to the determination that he was not a surgical candidate.

IDU, especially nonsterile injection practices, increases the risk of infective endocarditis caused by relatively uncommon environmental and ubiquitous pathogens, such as *Nocardia* spp. Hence, it is important for clinicians to suspect these organisms and obtain an appropriate history regarding injection practices. The primary treatment for endocarditis due to Nocardia is valvular surgery for source control. The reported mortality is high (60%) without valvular surgery. After source control is established, prolonged antibiotic therapy with multiple drugs is recommended, although the optimal duration is unknown.

## Data Availability

Data sharing is not applicable to this case report as no datasets were generated or analyzed during the current study.

## Abbreviations

PWUD: Persons who use drugs
MALDI-TOF MS: Matrix-assisted laser desorption time of flight mass spectrometry
IDU: Injection drug use
MICs: Minimum inhibitory concentrations
BPM: Beats per minute
mmHg: Millimeters of mercury
uL: Microliter
IU: International units
AV: Aortic valve
TMP-SMX: Trimethoprim-sulfamethoxazole
DSA: Digital subtraction angiography
LHC: Left heart catheterization
CRRT: Continuous renal replacement therapy
